# A case report of percutaneous direct injection of *N*-butyl-2-cyanoacrylate (NBCA) to treat a pancreatic duodenal stump leak after a simultaneous pancreas and kidney transplantation

**DOI:** 10.1186/s40792-021-01219-2

**Published:** 2021-06-08

**Authors:** Chisato Shirakawa, Masaaki Watanabe, Tsuyoshi Shimamura, Yasuyuki Koshizuka, Norio Kawamura, Ryoichi Goto, Takeshi Soyama, Daiki Iwami, Kiyohiko Hotta, Akinobu Taketomi, Daisuke Abo

**Affiliations:** 1 Department of Gastroenterological Surgery, Faculty of Medicine and Graduate School of Medicine, Hokkaido University, Kita-15 Nishi-7, kita-ku, Sapporo, 060-8638 Japan; 2 Department of Transplant Surgery, Faculty of Medicine and Graduate School of Medicine, Hokkaido University, Kita-15 Nishi-7, kita-ku, Sapporo, 060-8638 Japan; 3 Division of Organ Transplantation, Hokkaido University Hospital, Kita-15 Nishi-7, kita-ku, Sapporo, 060-8638 Japan; 4 Department of Diagnostic and Interventional Radiology, Hokkaido University Hospital, Kita-15 Nishi-7, kita-ku, Sapporo, 060-8638 Japan; 5 Department of Urology, Hokkaido University Hospital, Kita-15 Nishi-7, kita-ku, Sapporo, 060-8638 Japan

**Keywords:** Simultaneous pancreas and kidney transplantation, Duodenal stump leakage on pancreas graft, Direct injection of *N*-butyl-2-cyanoacrylate (NBCA)

## Abstract

**Background:**

Simultaneous pancreas and kidney transplantation (SPK) is a treatment option for patients with end-stage renal disease due to type 1 diabetes mellitus. We report a patient with a refractory fistula due to leakage from the duodenal stump of the pancreas graft after an SPK with bladder drainage who was successfully treated with a percutaneous direct injection of *N*-butyl-2-cyanoacrylate (NBCA).

**Case presentation:**

A 60-year-old female with a 33-year history of type 1 diabetes mellitus and a 10-year history of renal replacement therapy underwent an SPK in 2015. At the time of transplantation, an abdominal aortic aneurysm with a high risk of rupture was treated by a Y-graft replacement prior to the SPK. Bladder drainage of the pancreas graft was chosen to avoid a vessel graft infection. The patient’s postoperative course was uneventful. The patient was discharged on postoperative day 93 with good-functioning pancreas and kidney grafts. One and a half years after the operation, the patient was found to have acute graft pancreatitis and a leak from the duodenal stump of the pancreas graft due to a paralytic neurogenic bladder. The insertion of an indwelling catheter into the bladder and the endoscopic-guided insertion of a catheter into the graft pancreatic duct through the duodenum/bladder anastomosis did not result in the closure of the fistula. Therefore, NBCA was injected at the site of the leak point using CT-guided technique. The fistula was completely closed immediately after the injection, with no recurrences of leaks.

**Conclusions:**

A percutaneous direct injection of NBCA is one of the treatment options to treat intractable fistulas.

## Background

Simultaneous pancreas and kidney transplantation (SPK) is a treatment option for patients with chronic renal failure due to type 1 diabetes mellitus. However, the duodenal stump of the pancreas graft may leak, requiring complicated treatments. We present a patient with a refractory fistula due to leakage from the duodenal stump of the pancreas graft after SPK with bladder drainage who was successfully treated using percutaneous direct injection of *N*-butyl-2-cyanoacrylate (NBCA).

## Case presentation

**Fig. 1:**
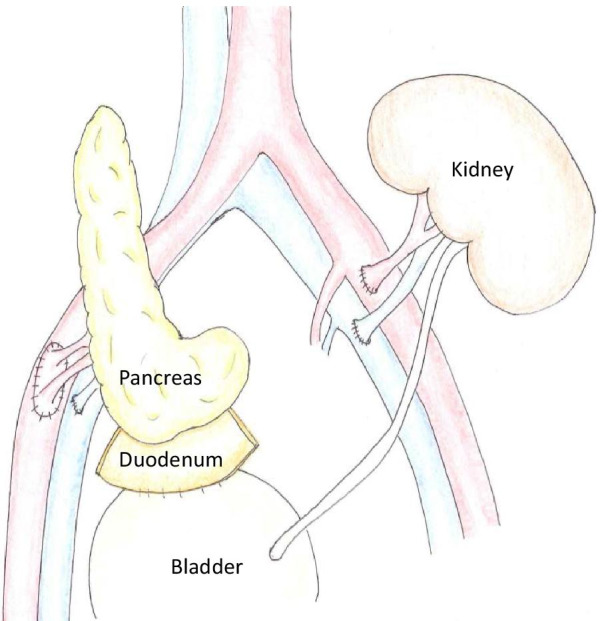
Surgical schema in SPK. Bladder-drained pancreas transplantation

**Fig. 2:**
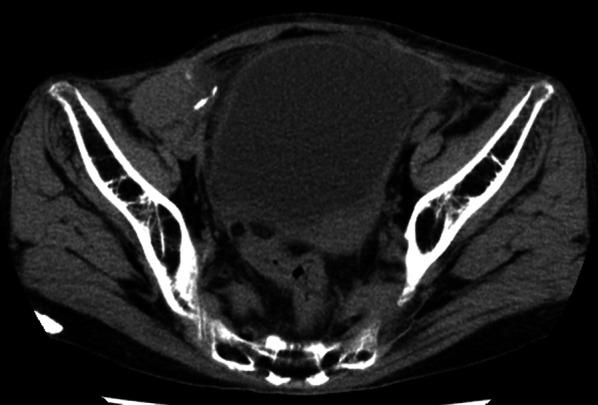
Abdominal computed tomography. A tense bladder is revealed on computed tomography

**Fig. 3:**
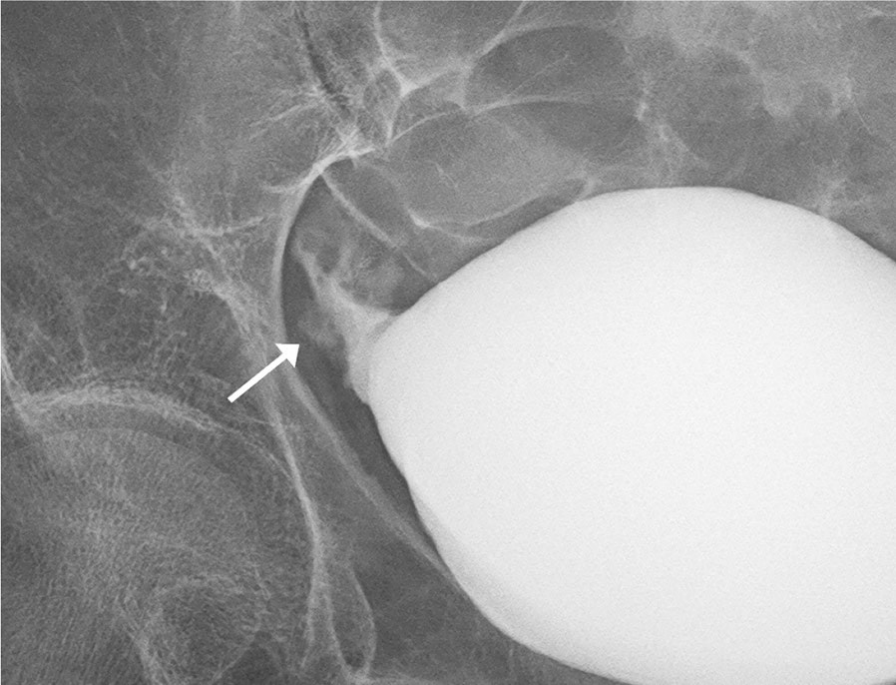
Cystogram. A minor leakage from the duodenal stump of the pancreas graft can be seen on the cystogram

**Fig. 4:**
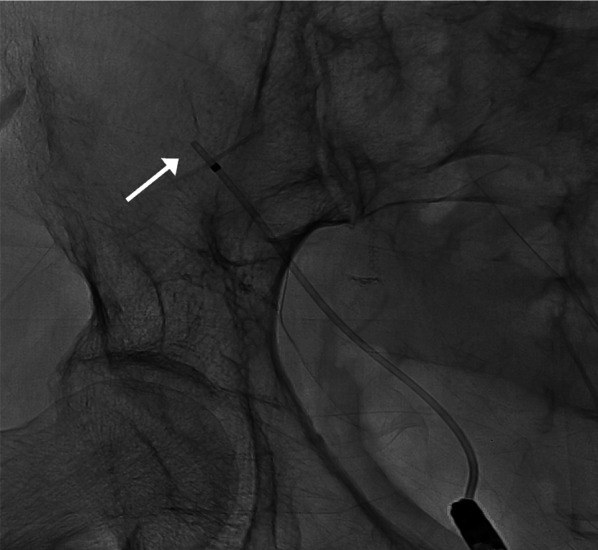
Endoscopic pancreatic catheter insertion. A pancreatic catheter is endoscopically inserted into the transplanted pancreas duct using the transurethral technique

**Fig. 5:**
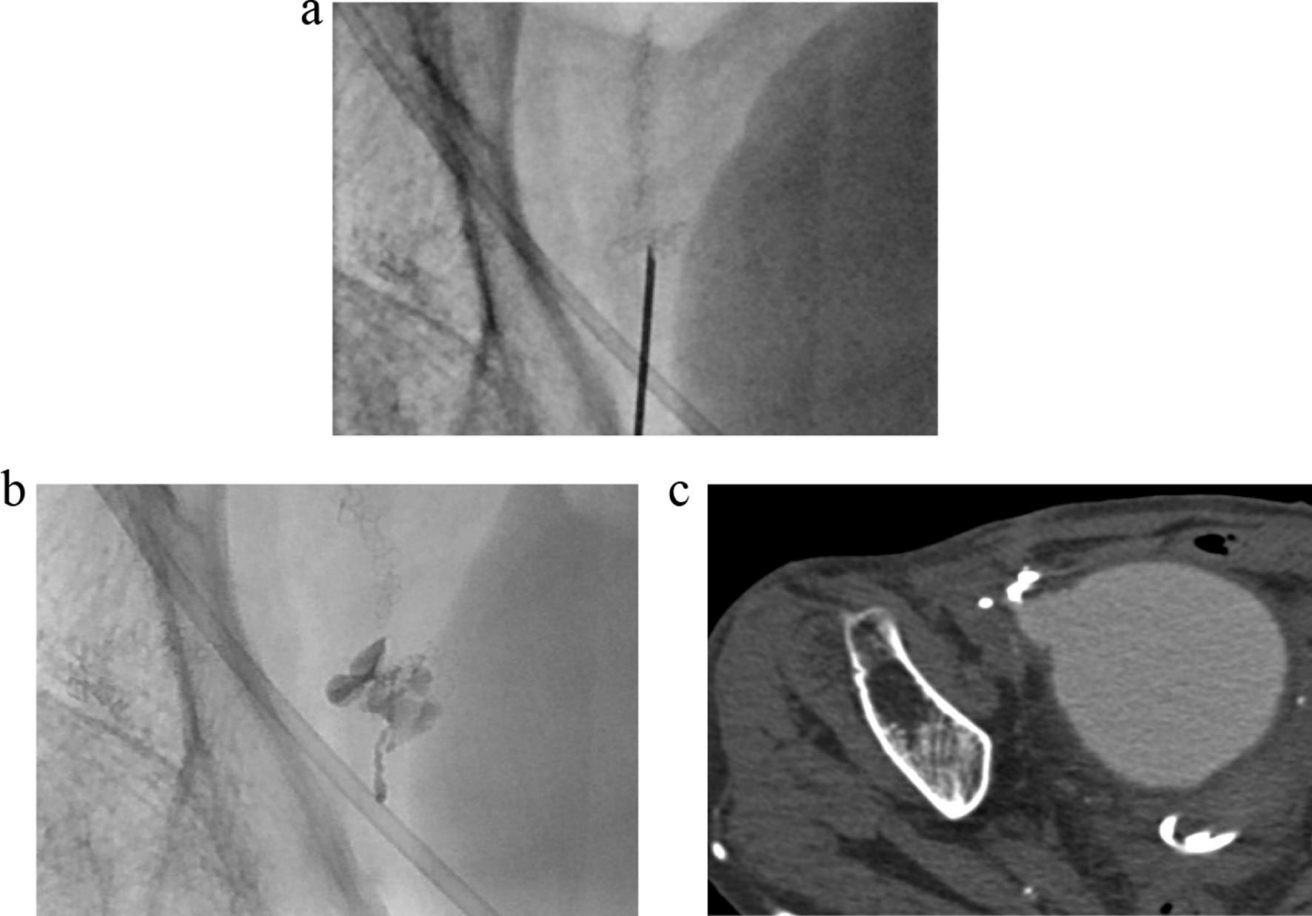
Percutaneous direct injection of *N*-butyl-2-cyanoacrylate (NBCA). **a** A 22-G needle tip is percutaneously advanced under CT-guided fluoroscopy to the site of the leak using the intersection of the surgical staples (arrow) as a marker. **b** Immediately after the injection of 0.1 ml of 50% NBCA, a fluoroscopic image shows excellent retention of the NBCA cast at the leak site (arrow). **c** A CT image obtained immediately after the injection of 0.1 ml of 50% NBCA shows excellent retention of the NBCA cast at the leak site (arrow)

A 60-year-old female with a 33-year history of type 1 diabetes mellitus and a 10-year history of renal replacement therapy underwent an SPK in 2015 (Fig. 1). One and a half years after the transplantation, the patient presented with lower abdominal pain. Laboratory data revealed that the white blood cell count (4000/μl), serum CRP (< 0.02 mg/dl), and blood glucose (112 mg/dl) were normal. However, the serum amylase (174 U/L; normal range: 44–132 U/L) and lipase (88 U/L; normal range: 13–49 U/L) were elevated, indicating acute pancreatitis of the pancreas graft. A CT revealed urinary retention (Fig. 2), and a cystogram showed a minor leak from the duodenal stump of the pancreas graft (Fig. 3). The patient’s abdominal pain was thought to be caused by the pancreatitis in addition to the leak from the duodenal stump into the abdominal cavity. In addition to the treatment with antibiotics and protease inhibitor, a 20 Fr indwelling catheter was inserted to reduce the pressure inside of the bladder. The excretion of 900 mL of residual urine indicated that the increased intravesical pressure due to a neurogenic bladder induced the leak from the duodenal stump and the graft pancreatitis. The fistula did not close over 8 weeks of bladder catheter insertion. To reduce tissue injury by pancreatic exogenous fluid at the site of the leak, an endoscopic pancreatic catheter (Cliny5Fr α ENBD) was placed into the transplanted pancreas duct through the bladder (Fig. 4). However, the fistula did not completely heal. Therefore, after obtaining informed consent from the patient, NBCA (Histoacryl®, Aesculap, Tuttlingen, Germany) diluted to 50% with iodized oil (Lipiodol, Guerbet, Tokyo, Japan) was percutaneously directly injected at the site of the leak under CT-guided fluoroscopy (Fig. 5). The fistula was closed immediately after the injection, with no recurrences of leaks.

## Conclusions

In cadaveric pancreas transplantations, the pancreas graft is ectopically transplanted into the retroperitoneal space or at the iliac fossa, and the duodenum of the pancreas graft is anastomosed to the small bowel (enteric drainage; ED) or the bladder (bladder drainage; BD) to provide pancreatic exocrine drainage. The ED of pancreatic exogenous fluid is the more physiologic technique and has a lower risk of urinary tract infections (UTI) [1, 2]. However, ED may lead to an intraabdominal abscess or peritonitis if anastomotic leaks occur. In contrast, the BD anastomosis can be performed in the retroperitoneal space. In addition, the function of the pancreatic graft can be monitored by measuring urinary amylase levels. However, BD may lead to metabolic acidosis, volume depletion secondary to sodium bicarbonate wasting, reflux pancreatitis, UTI, and hematuria [1]. Severe UTIs caused by multidrug-resistant bacteria can occur after BD-SPK [3], and the rate of urinary retention after renal transplantation has been reported to be 5.8% [4]. Therefore, the ED method is currently selected during pancreas transplantations in 90% of the transplant centers in Japan as recent developments in immunosuppressive therapies have decreased the risk of acute rejection, and ED has been reported to result in more favorable outcomes than BD [5–8]. Furthermore, a high rate of conversion surgery from BD to ED (20–25%) due to complications of UTI or hematuria has been reported [9].

This patient had a concomitant spindle-shaped abdominal aortic aneurysm with a diameter of 3.3 cm at the time of the SPK, and an annual growth of 0.5 cm indicated the requirement of treatment. We performed an artificial vascular graft replacement to the aneurysm prior to the pancreas and kidney implantations in order to avoid low blood flow to the grafts when the aneurysm replacement was performed after the SPK. The BD method was selected on the pancreas engrafting as it has a lower risk of intraperitoneal infection compared to ED. The vascular graft infection is one of the serious complications with high mortality and morbidity rate. Once infected, early mortality rate has been reported 24 to 45% [10–13], and infected artificial vascular graft needs to be completely removed with extra-anatomic bypass grafting.

Following the SPK with BD, our patient was followed up with monthly blood tests and urine amylase checks. One and a half years after the transplantation, the patient presented with lower abdominal pain, which we diagnosed as pancreatitis and leakage from the duodenal stump of the pancreas graft into the peritoneal cavity caused by urinary retention related to a neurogenic bladder. While a urodynamic study can be used to evaluate bladder function, we do not regularly perform this study in the posttransplant period [14]. There are currently no guidelines regarding the frequency of urodynamic studies. However, the evaluation of the bladder function in this patient may have prevented the pancreatitis or the duodenal stump leak.

Urinary drainage with an indwelling catheter is the standard first-line treatment for leaks from the duodenal stump after SPK with BD. Surgical repair is not always necessary to treat late-onset leakage or fistulas after SPK [2]. However, the indwelling catheter did not lead to a resolution of the fistula in this patient. As reported [15], we endoscopically inserted a pancreatic tube, which allowed for the drainage of the graft pancreatic juice, but did not resolve the leak in the duodenal stump of the pancreas graft. Given the fact that patient had not been suffering from the leakage during 18 months after the operation, the onset of the leakage from the duodenum stump might be mainly due to neurogenic bladder. Prolonged high pressure in the bladder may prevent blood supply to the stump of the duodenum. Poor blood supply and/or high level of amylase concentration generally impedes healing fistula [16]. Finally, we percutaneously and directly injected 50% NBCA at the site of the leak, which resulted in the complete closure of the fistula without any adverse effects. NBCA is a tissue adhesive and is usually mixed with iodized oil. It is a tissue monomer that instantly polymerizes upon contact with body fluids. NBCA is used to stop bleeding and close postoperative fistulas [17]. In addition, embolization using NBCA has been reported for the treatment of esophageal gastric varices, suture failures, pancreatic fluid leaks, and bile leaks [17–21]. NBCA has been approved to be used as an endoscopic hemostatic agent for gastric varices and as a tissue adhesive for the skin. The injections of NBCA may induce local pain, infection, and even embolism when injected incorrectly into blood vessels. All procedures used in the NBCA injection were approved by the Ethical Committee of Hokkaido University Hospital, and written informed consent was obtained from the patient before the treatment with NBCA. This case demonstrates that NBCA can be used to treat refractory duodenal stump leakages. To the best of our knowledge, this is the first report of successful percutaneous direct injection of NBCA to treat refractory fistulas.

In conclusion, leaks from the duodenal stump of a pancreas graft can occur after an SPK. The percutaneous direct injection of NBCA to the site of the leak can be used to treat this complication.

## Data Availability

Not applicable.

## Abbreviations

BD: Bladder drainage
ED: Enteric drainage
NBCA: *N*-Butyl-2-cyanoacrylate
SPK: Simultaneous pancreas and kidney transplantation
